# Hemodynamics of the VenusP Valve System™—an *in vitro* study

**DOI:** 10.3389/fmedt.2024.1376649

**Published:** 2024-05-02

**Authors:** Huang Chen, Milad Samaee, Michael Tree, Lakshmi Dasi, Ajit Yoganathan

**Affiliations:** ^1^ Department of Biomedical Engineering, Georgia Institute of Technology and Emory University, Atlanta, GA, United States; ^2^ Department of Mechanical Engineering, University of Nevada, Las Vegas, NV, United States; ^3^ Corvid Technologies LLC, Mooresville, NC, United States

**Keywords:** transcatheter pulmonary valve replacement, VenusP Valve System, hemodynamics, Particle Image Velocimetry, leaflet kinematics, Reynolds shear stress

## Abstract

This study aims to evaluate the fluid dynamic characteristics of the VenusP Valve System™ under varying cardiac outputs *in vitro*. A thorough hemodynamic study of the valve under physiological cardiac conditions was conducted and served as an independent assessment of the performance of the valve. Flow fields downstream of the valve near the pulmonary bifurcation were quantitatively studied by two-dimensional Particle Image Velocimetry (PIV). The obtained flow field was analyzed for potential regions of flow stasis and recirculation, and elevated shear stress and turbulence. High-speed en face imaging capturing the leaflet motion provided data for leaflet kinematic modeling. The experimental conditions for PIV studies were in accordance with ISO 5840-1:2021 standard, and two valves with different lengths and different orientations were studied. Results show good hemodynamics performance for the tested valves according to ISO 5840 standard without significant regions of flow stasis. Observed shear stress values are all well below established hemolysis limits.

## 1 Introduction

Pulmonary stenosis (PS) and pulmonary regurgitation (PR) are serious conditions associated with pulmonary valve incompetence. If left untreated, significant complications including heart failure will occur. Although the first transcatheter pulmonary valve was successfully implanted in a patient two decades ago (1, 2), these early-generation balloon-expandable valves can only be used in a failed conduit or bioprosthetic valve (3–5). However, PS and PR patients with native anatomy account for over 80% of the total cases (3). Large right ventricle outflow tract (RVOT) sizes and variations in native anatomy make a self-expandable valve a more attractive option. Currently, the only FDA-approved product is the Harmony Valve (6, 7) (Medtronic, Minneapolis, NN), with a few more products in clinical trials (8–10). The VenusP-Valve System™ is designed to replace the pulmonary heart valve with an artificial valve using a minimally invasive transcatheter approach. VenusP-Valve System™ is used for the treatment of moderate or severe (≥3+) pulmonary regurgitation with or without stenosis in patients with native right ventricular outflow tracts, therefore reducing pulmonary regurgitation.

## 2 Materials and methods

To investigate hemodynamics, two 36 mm diameter VenusP Valve Systems™ of different lengths (30 and 25 mm) from Venus Medtech (Hangzhou) Inc. were shipped and tested by the Cardiovascular Fluid Mechanics Laboratory (CFM Lab) at the Georgia Institute of Technology. The valve diameter tested is the largest in the VenusP Valve System™ lineup. The VenusP Valve System™ is available in sizes that cover 85% of the population. The serial numbers of the tested valves are:

(1) L36P, P36-30 (30 mm in length)
(2) L36P, P36-25 (25 mm in length)

**Figure 1:**
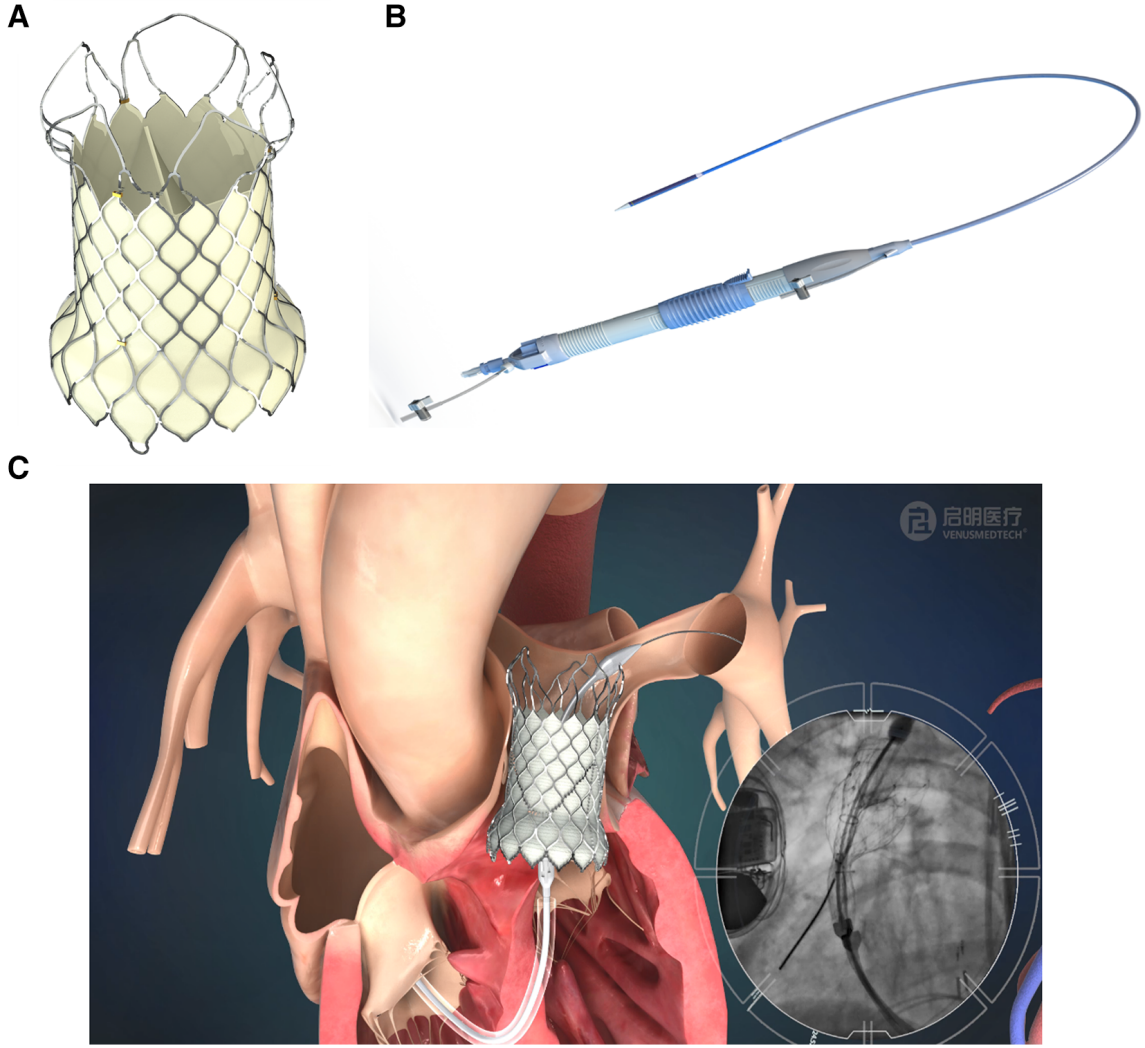
VenusP Valve System™. (**A**) Percutaneous Pulmonary Valve, (**B**) the delivery catheter system and (**C**) animation and angiogram showing the deployment of the valve.

The self-expandable VenusP Valve System™ is shown in Figure 1. The percutaneous pulmonary valve (PPV) consists of a self-expanding nitinol support frame with a tri-leaflet porcine pericardium tissue valve. The PPV is made of a single layer of porcine pericardium built in a tri-leaflet configuration. These are attached to a scalloped skirt on the inflow aspect of the valve using PTFE sutures. The valve is designed to treat RVOT dysfunction and specifically for the dilated outflow tracts to restore pulmonary valve conduit function. The self-expanding, multi-level frame is made of nitinol and has radiopaque markers attached to it.

The delivery system is shown in Figure 1B, and an illustration of the deployed valve in the pulmonary artery is shown in Figure 1C. The fluoroscopy image inset in Figure 1C shows the deployment process.

**Figure 2:**
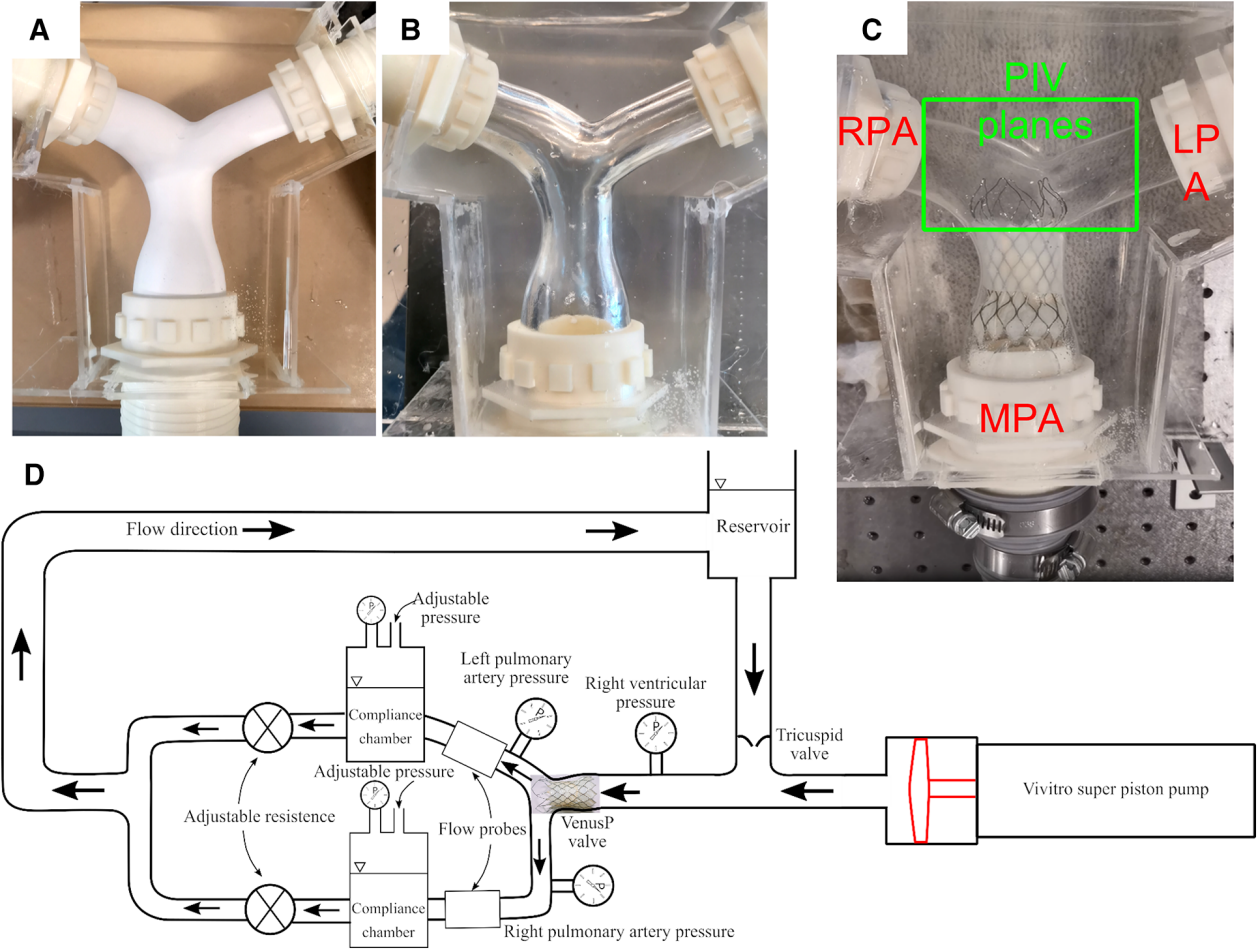
(**A**) Cast PDMS chamber with the soluble core, (**B**) with the core removed, and (**C**) with VenusP Valve System™ deployed. (**D**) The right heart simulator is capable of generating physiological flow and pressure.

The VenusP Valve System™ was inserted into a cast Polydimethylsiloxane (PDMS) pulmonary valve (PV) chamber, whose geometry was based on median patient anatomy with dilated PVs provided by Venus Medtech (Hangzhou) Inc. The chamber was designed and made by the CFM Lab at Georgia Tech out of a PDMS block. The geometry of the pulmonary bifurcation is shown in Figure 2. It consists of an RVOT as the inlet and a right pulmonary artery (RPA) and left pulmonary artery (LPA) as the outlets. The RPA and LPA are separated by approximately 120°. Detailed geometries are shown in the Supplementary Material S1. The flow split between LPA and RPA was set to be 45% and 55% of the inlet flow, respectively, which is considered the normal distribution in the literature (11, 12). The valves were tested under cardiac outputs od 2.5 L/min and 5 L/min, which were within ISO 5840 recommended range of 2–7 L/min.

Pictures of the casting process and the flow chamber are shown in Figures 2A,B. The flow chamber was constructed by first manufacturing a soluble core (Plaster of Paris) that conformed to the RVOT and pulmonary artery geometry. Then, PDMS silicone rubber was poured into a molding box containing the soluble core. After the PDMS was cured, the soluble core was washed out. Details about the casting process can be found in (13–15). The chamber provides optical access to the valve (Figure 2) for capturing leaflet motion and flow visualization. Figure 2C shows the flow phantom with the deployed VenusP Valve System™. A thin flexible silicone sleeve was used between the rigid flow chamber and the deployed value to prevent paravalvular leakage.

### 2.1 Right heart simulator setup

The Georgia Tech Right Heart Simulator, shown by the schematic in Figure 2D, was used as a pulsatile flow loop to provide anatomically realistic pressures and flows to the VenusP Valve System™ mounted in the flow chamber. The loop consisted of a fluid reservoir, a mechanical tricuspid valve, a Vivitro super piston pump (Vivitro Labs, Victoria, BC, Canada), the pulmonary valve chamber, and two compliance chambers followed by two resistance elements. These afterload elements were manually adjusted to generate physiological flow and pressure waveforms. Specifically, compliance is varied by changing the water level of the chamber while the resistance is adjusted by opening or closing the valve (16). The piston pump system was driven by internally stored physiological waveforms (Physio-70) from the pump manufacturer and was synchronized with the imaging system. The valve's desired flow and pressure characteristics were controlled by tuning piston pump stroke volume, compliance, and resistance levels. The Newtonian working fluid was a 36% by-volume glycerin solution in water to mimic the dynamic viscosity of blood (*µ *= 3.6 × 10^−3 ^*Pa•s* at 37°C, measured by an NDJ-5S viscometer). The working fluid also provided reduced optical distortions through the PDMS chamber as it matched the refractive index of PDMS closer than saline. The LPA and RPA flows were monitored using two inline ultrasonic flow probes with amplifier boxes (Model ME 20PXL, Transonic Inc., Ithaca, NY). The flow rate in the MPA was obtained by summing the flow rates measured by LPA and RPA, under the assumption that the model was rigid. Absolute pressure was measured by Millar pressure catheters (Millar, Inc, Houston, TX) on both the ventricular and pulmonary artery sides (main PA distal to valve). The flow and pressure signals were acquired by USBDAQ-6218 (National Instruments Corp, Austin, TX) connected to a PC, and controlled using a custom LabView program. Flow and pressure data were acquired at 1,000 Hz using the data acquisition system. All data was collected for 15 cycles and averaged before analysis. From the data acquired, the mean and peak systolic pressure drop (Δ*P_mean_*, Δ*P_peak_*) and effective orifice area (EOA) were calculated.

Pressure drop was calculated as the difference between right ventricular and pulmonary artery pressure:

(1) $$\Delta P_{mean}=\frac{1}{T}\int_{systole}\Delta Pdt$$

(2) $$\Delta P_{\;peak}=\max(\Delta P{)}_{systole}$$

The EOA was calculated from the pressure measurement using the following orifice equation:

(3) $$EOA=\frac{Q}{51.6\sqrt{\Delta P_{mean}}}$$

where *Q_rms_* is the root-mean-square (*rms*) flow rate during systole expressed in ml/s, Δ*P* is the mean systolic pressure drop in mmHg, and EOA is expressed in cm^2^. In this study, EOA was computed using the updated ISO definition, which uses only the positive systolic gradient period as the duration for *Q_rms_* and Δ*P_mean_*.

### 2.2 High-speed en face imaging

To examine leaflet kinematics and provide leaflet position and motion data for subsequent simulations, en face high-speed videos were recorded during experiments for both valves at both orientations. The leaflets were illuminated by LED work lamps and the images were recorded by a high-speed monochromatic camera (Photron FastCam SA3, 1,024 × 1,024 pixels) at 2,000 frames per second. Still images from the en face videos at peak systole were used to calculate the geometric orifice area (GOA), which is the anatomical area of the aortic valve orifice.

### 2.3 PIV setup

**Figure 3:**
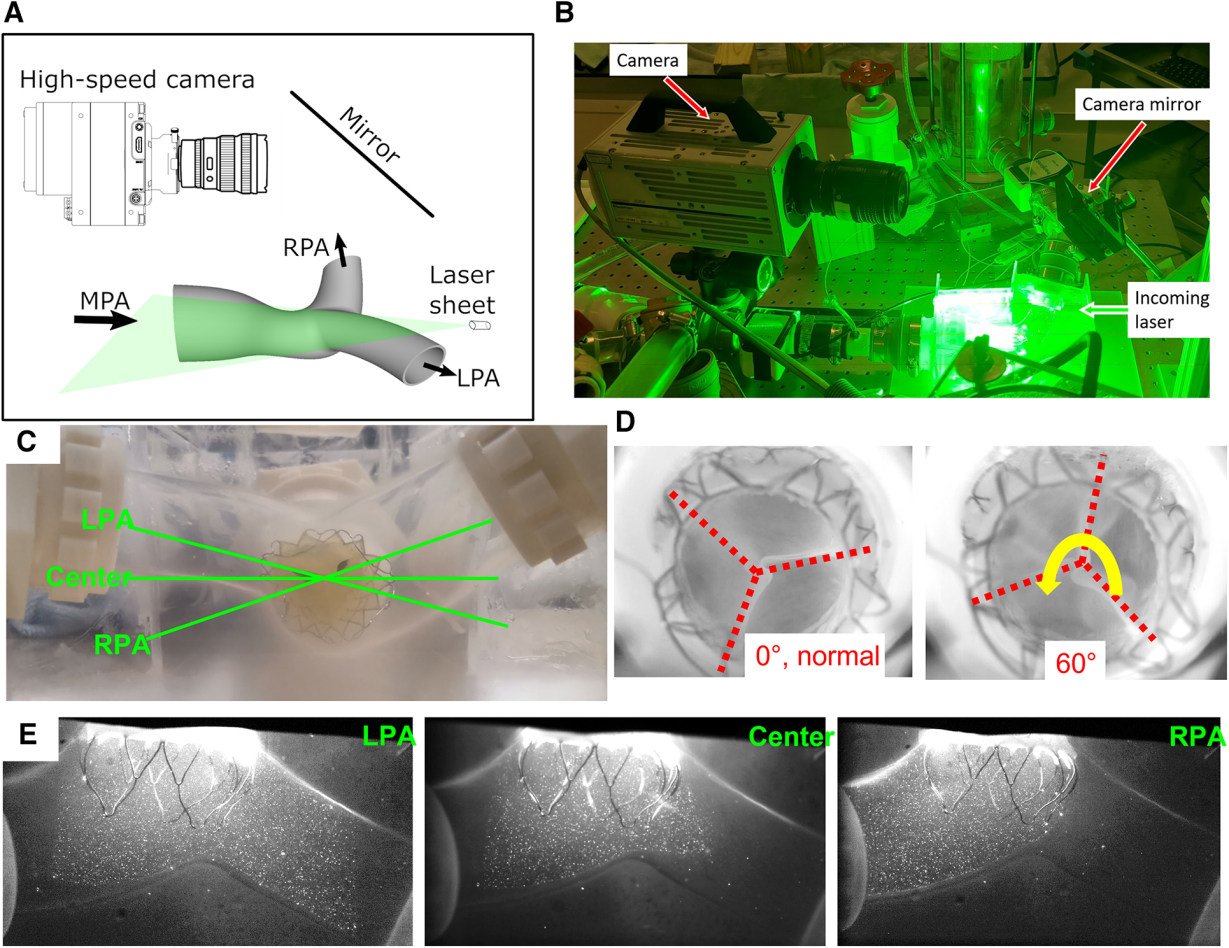
PIV experimental setup. (**A**) A sketch and (**B**) a photo of the imaging setup for PIV experiments. (**C**) The imaging planes. (**D**) Two different valve orientations. (**E**) Sample particle images from three image planes.

The current study focused on quantitative flow field evaluations of the downstream vicinity of the longer valve (P36-30) using Particle Image Velocimetry (PIV). The valves were mounted on the right heart simulator described in *Right heart simulator setup* and shown in Figures 2C,D. The PIV setup is shown in Figures 3A,B. A pulsed Nd:YLF laser source (Photonics Industries, DM40-527) was used to create a thin laser sheet of thickness∼1 mm using adjustable sheet optics provided by LaVision (Article number 1108405, LaVision, Göttingen, Germany). The laser sheet was diverted to illuminate the region of interest using a series of laser mirrors and translation stages, enabling easy position adjustments. A Complementary Metal-Oxide-Semiconductor (CMOS) camera (Photron FastCam SA3, 1,024 × 1,024 pixels) with a 60 mm Micro-Nikkor lens, spacer rings, and a long-pass filter (cut-off at 540 nm) was used to capture images of the scattered light from the seeding particles. The flow was seeded with fluorescent particles (PMMA, coated with Rhodium-B dye) of nominal diameter 1–20 μm. PIV images were acquired using DaVis 7.2 (LaVision, Göttingen, Germany). The camera was mounted horizontally, and the images were acquired through a 45-degree mounted mirror (Figures 3A,B). Velocity vectors were obtained across the entire field of view by mathematically cross-correlating two particle images separated by a known time interval (Δ*t *= 0.5–1 ms).

The three imaging planes were determined based on a discussion with Venus MedTech Inc., shown in Figure 3C. The LPA and RPA planes are aligned with their corresponding arteries, while the center plane is horizontal to the model. All the planes intersect with the valve center. Samples of the PIV images are shown from all three planes (Figure 3E). Due to the refractive index similarities between the PDMS chamber and the water-glycerin working fluid, there is no visible distortion near the boundaries. The imaging setup slightly differs from the three parallel planes recommended in ISO 5840-1-2021, Figure H.3 (1). Since the left and right PAs are at an angle with the main PA, the top and bottom parallel planes have very limited field of view. On the contrary, the current three intersecting plane setup generates a more comprehensive picture of the flow downstream of the valve. Furthermore, it enables the examination of the flow downstream in the distal RPA and LPA. To address the concern of a misaligned deployment of the valve, the valve was also mounted 60° away from its normal deployment orientation (Figure 3D), and the PIV experiments were repeated. The two distinct orientations provide a more complete understanding of the flow past the valve.

A calibration procedure was conducted to convert the calculated pixel displacements to physical space. Calibration involved acquiring images of a measured grid pattern aligned with the laser sheet to compute a pixel displacement to physical space conversion and correct any optical distortions. The PIV images acquired were phase-locked to the piston pump allowing for precise timing within the cardiac cycle. At each phase, 200 image pairs were acquired for ensemble averaging. Data at 20 phases throughout the cardiac cycle was acquired (15 during systole separated by 23 ms and 5 during diastole separated by 100 ms, since most flow features are expected to be observed during systole). This data acquisition method provided highly accurate mean flow features and statistics at the selected phases.

**Figure 4:**
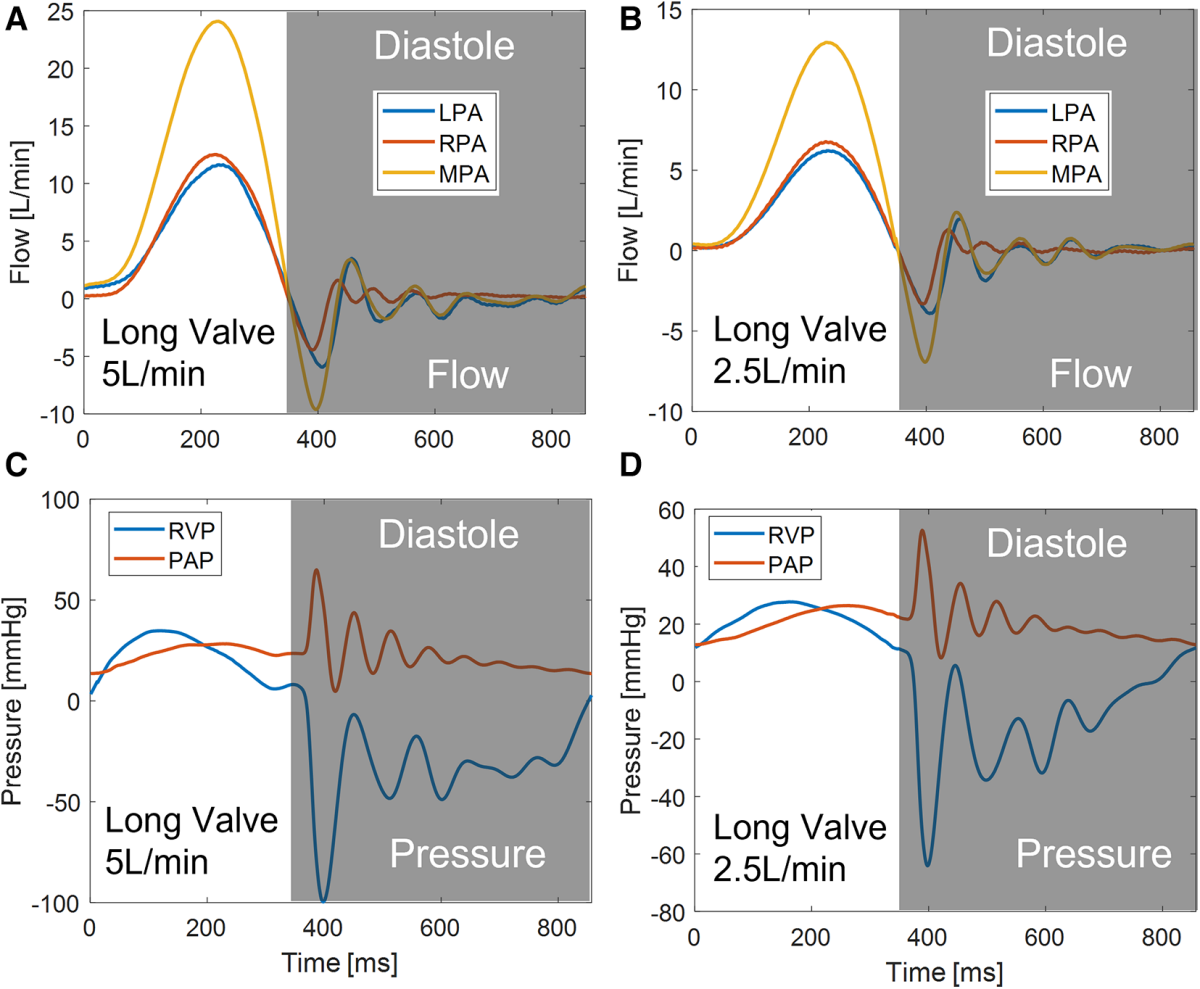
Flow and pressure for the long valve (P36-30) under two different flow rates.

Experiments were carried out at both 5 L/min and 2.5 L/min, at 70 beats per minute and 35% of systolic duration (Figure 4). For both flow rates, the peak-systolic and end-diastolic pressure difference was kept at 15 mmHg, the recommended normotensive value by ISO 5840 standard. All the obtained datasets are summarized in Table 1. Due to extreme laser reflections off the valve stent (Figure 3E), masking of valve components and/or very slight adjustment of the laser plane was necessary to improve data yield. The PIV images were post-processed using DaVis 7.2 (LaVision GmbH, Göttingen, Germany) for all the conditions acquired.

**Table 1:** Datasets acquired for all the valves and test conditions.

Valve	En face video	Phase-locked PIV
P36-30 mm	Two takes at 2,000 fps, 4 cardiac cycles each for: • Two orientations (normal, 60°) • Two flow rates (5 L/min, 2.5 L/min).	15 phases at systole and 5 at diastole, 200 image pairs for each phase for: • Two orientations (normal, 60°) • Two flow rates (5 L/min, 2.5 L/min) • Three planes (LPA, RPA, center)
P36-25 mm	Two takes at 2,000 fps, 4 cardiac cycles each for: • One orientation (normal) • Two flow rates (5 L/min, 2.5 L/min) • Three planes (LPA, RPA, center)	• None

### 2.4 PIV data analysis

Processed PIV images provide velocity vector fields in the plane of interest. The system used in the present experiments was a two-component PIV system, which provided a vector resolution of 0.87 mm with a final interrogation window size of 48 px^2^ with 75% overlapping. After the velocity vectors were computed using DaVis 7, the data were exported to a workstation. Later a custom Matlab (MathWorks, Inc., MA) program was used to calculate additional derived quantities from the velocity fields. Using the 200 velocity vector fields obtained at each time point at every experimental condition, an ensemble-averaged mean velocity vector and other flow statistics fields were obtained.

By subtracting the mean velocity field from the instantaneous velocity field, a fluctuating velocity field was obtained. This is mathematically represented as:

(4) $$u^{\prime }=U-\bar{U}$$

where $u^{\prime }$ is the fluctuating velocity, *U* is the instantaneous velocity and $\bar{U}$ is the mean velocity. The fluctuating velocity field characterizes the levels of variation in the flow fields from cycle to cycle. The following additional quantities were also calculated:

*Viscous shear stress*: Viscous shear stresses capture the effect of shearing between adjacent layers of fluid, which is a physical force exerted by the fluid on suspended blood cells. The in-plane viscous shear stress (*VSS*) that can be calculated from the 2D PIV measurements is defined as:

(5) $$VSS=\mu \left(\frac{\partial V}{\partial X}+\frac{\partial U}{\partial Y}\right)$$

*Principal Reynolds shear stress*: The principal Reynolds shear stress has been well correlated to blood cell damage and is used to predict potential regions of hemolysis. The principal Reynolds shear stress (*RSS*) is defined as:

(6) $$RSS=\rho \sqrt{{\left(\frac{\bar{u^{\prime }u^{\prime }}-\bar{v^{\prime }v^{\prime }}}{2}\right)}^{2}+{\left(\bar{u^{\prime }v^{\prime }}\right)}^{2}}$$

## 3 Results

### 3.1 Leaflet kinematics

**Figure 5:**
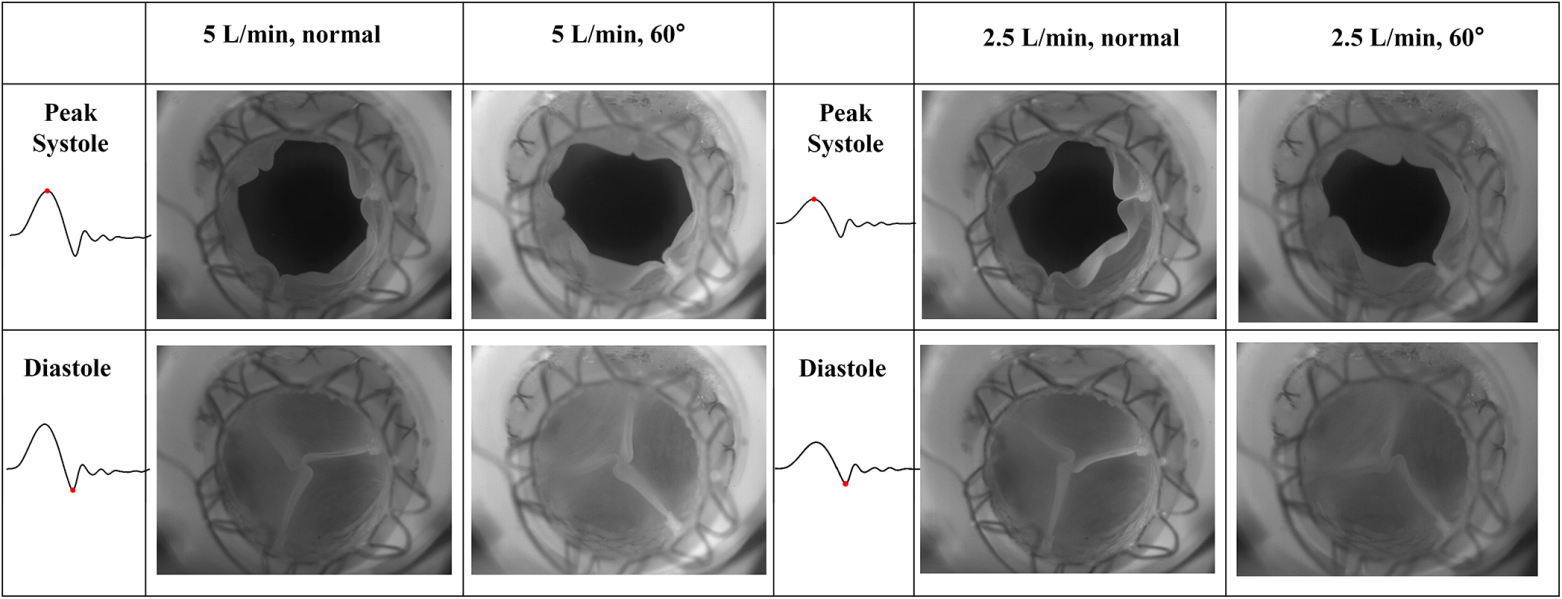
High-speed en face imaging of leaflet motion at peak systole and diastole for normal and 60°orientations, and 5 L/min and 2.5 L/min. The time points corresponding to the various flow phases are shown in the insets in the corresponding figures.

**Figure 6:**
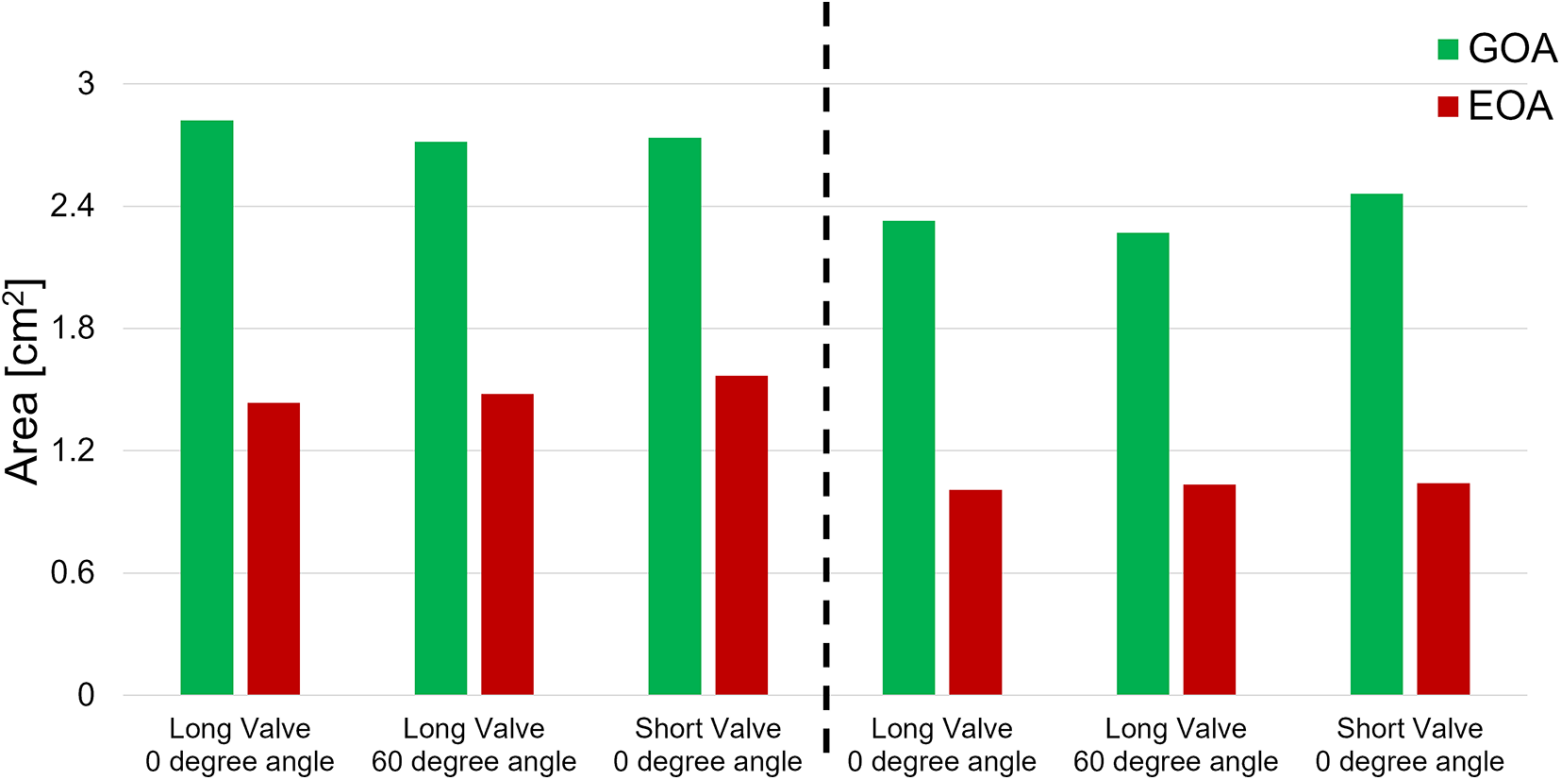
Geometric and effective orifice area of various valve orientations and various cardiac outputs.

The goal of this section is to evaluate the kinematics of the leaflets under different mounting and flow conditions. High-speed en face videos of leaflet motion for P36-30 valve are shown in Figure 5. Two phases in the cardiac cycles are discussed—peak systole, when the valve has the largest opening, and diastole, when the valve is fully closed. The time points corresponding to the various flow phases for the flow visualization studies are shown in the insets in the corresponding subfigures. The geometric orifice area (GOA) calculated based on the en face videos are shown in Figure 6 and tabulated in Table 2, for both valves tested.

**Table 2:** Hemodynamic results for VenusP Valve System™ at various valve orientations and various cardiac cycle.

Cardiac output (L/min)	Valve type	Valve orientation	Mean ΔP (mmHg)	EOA (cm^2^)	GOA (cm^2^)
2.5	P36-30 mm	Normal	4.66	1.01	2.32
		60^o^	3.92	1.03	2.27
	P36-25 mm	Normal	4.15	1.04	2.46
5.0	P36-30 mm	Normal	7.28	1.43	2.82
		60^o^	7.37	1.48	2.71
	P36-25 mm	Normal	7.30	1.57	2.74

At peak systole, the valves are fully open, and peak flow is reached. The GOA for P36-30 valve at the normal orientation is 2.82 cm^2^, while it reduces slightly to 2.71 cm^2^ when the valve is rotated 60°. The shape of the valve opening for the 60° case is the same as the 0° valve rotated 60° in the counter-clockwise direction. The different orientation does not affect the kinematics of the leaflets near peak systole. When the flow rate is reduced to 2.5 L/min, the GOA is smaller for both valve orientations, which indicates that the flow rate does impact the valve opening.

At diastole, the valve is closed, and the 60° counter-clockwise rotation of the valve can be easily identified from the second row of Figure 5. All valves tested closed properly for all the cases with no leakage. There is slight pin-wheeling upon leaflet closure, but it should not affect the valve's performance (Figure 6 and Table 2). It should also be noted that the pin-wheeling may be caused by the rigid chamber in the current experiments. A patient's artery is more flexible and may allow the valve to open wider upon mounting.

### 3.2 Hemodynamic characterization

The hemodynamic characterization studies for the VenusP Valve System™ are shown in Table 2 and Figure 6. All the curves are synchronized with the motion of the piston pump, with time zero to be the start of a push stroke. At the low flow rate/cardiac output (2.5 L/min), the mean *ΔP* and EOA values are smaller when compared to the higher cardiac output conditions (5 L/min). Among all three cases (various valve lengths and orientations), the maximum difference in peak pressure and peak flow values was less than 0.5%.

We did not notice a significant difference between GOA values in different cases. Similarly, the difference between EOA values in different cases was small (Figure 6 and Table 2). However, the EOA value seems to be low compared with the GOA. Since the pressure downstream of the valve was measured in the RPA, compared to what was usually measured just downstream of the valve, there is a significant pressure loss due to the flow through the pulmonary bifurcation. Since the pressure gradient across the pulmonary valve is small, the pressure loss added a non-negligible value to the mean Δ*P* and hence the calculated EOA. We expected the EOA to be closer to the GOA when the pressure is measured just downstream of the valve.

### 3.3 Flow field downstream of the valve

**Figure 7:**
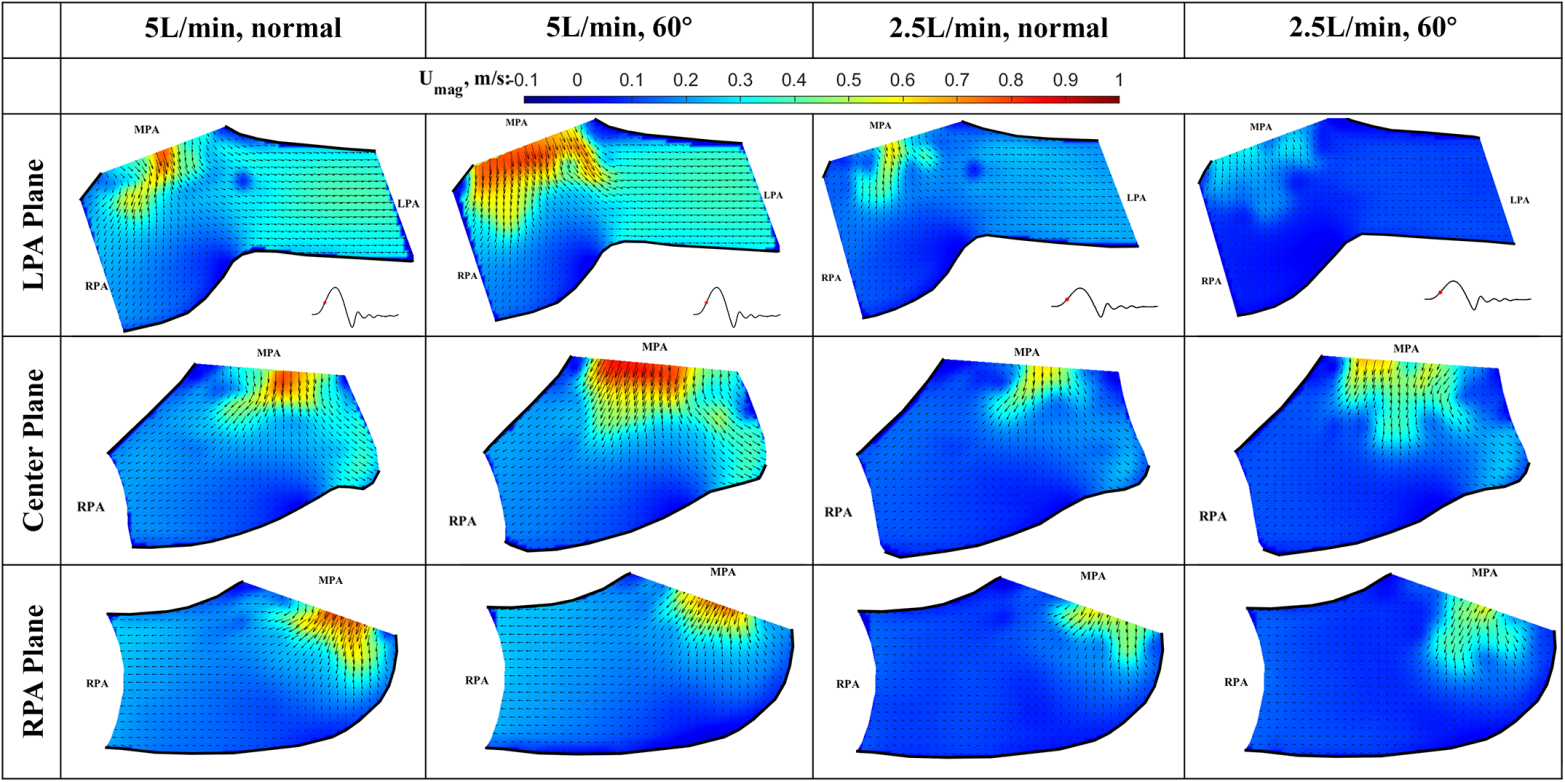
In-plane vector fields over contours of velocity magnitude during the acceleration phase for LPA/center/RPA planes (top/middle/bottom rows) for different flow rates and valve orientations. The time point corresponding to the flow phase in the cardiac cycle is shown in the insets in the first row.

**Figure 8:**
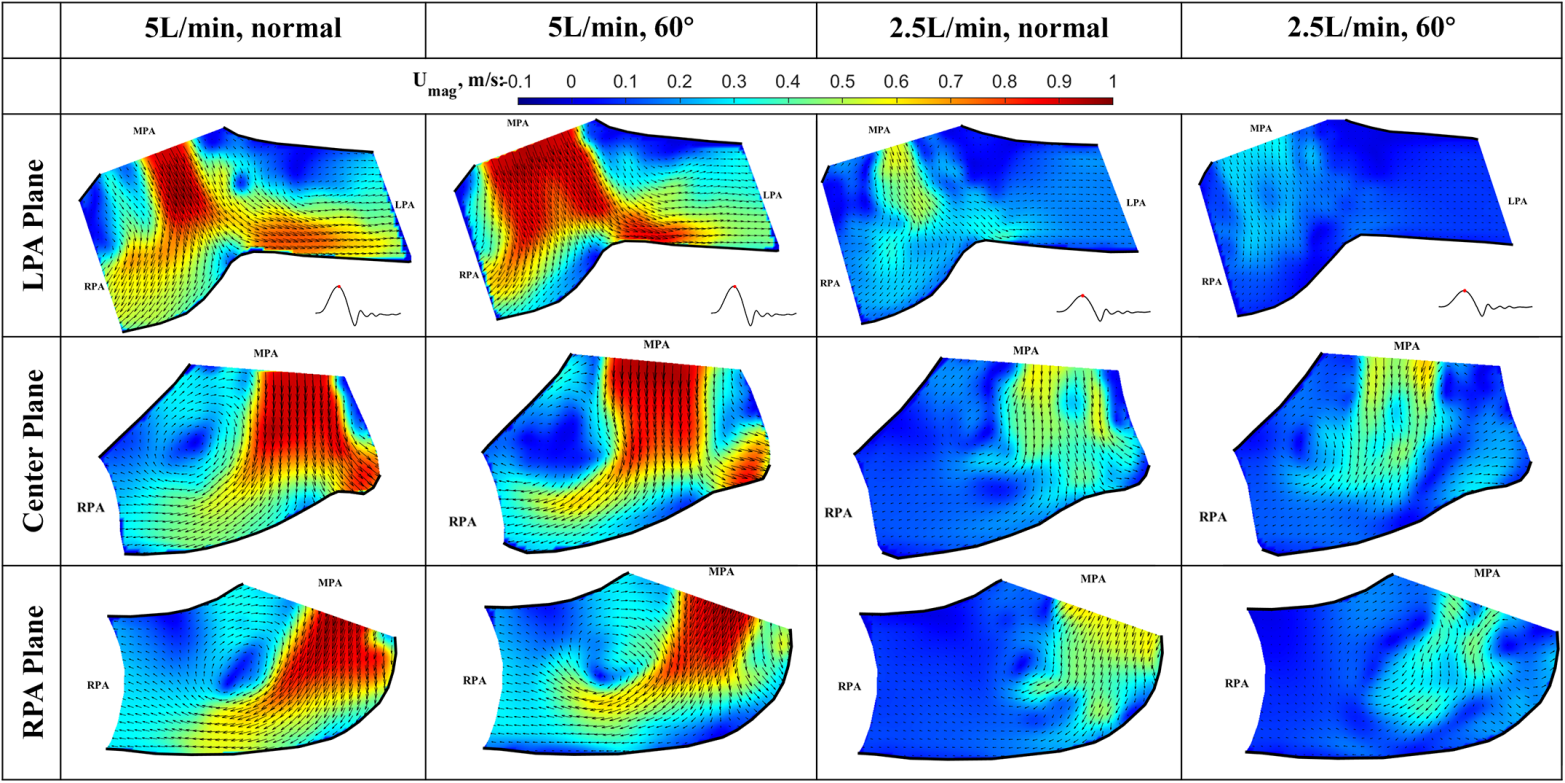
In-plane vector fields over contours of velocity magnitude at peak systole for LPA/center/RPA planes (top/middle/bottom rows) for different flow rates and valve orientations. The time point corresponding to the flow phase in the cardiac cycle is shown in the insets in the first row.

**Figure 9:**
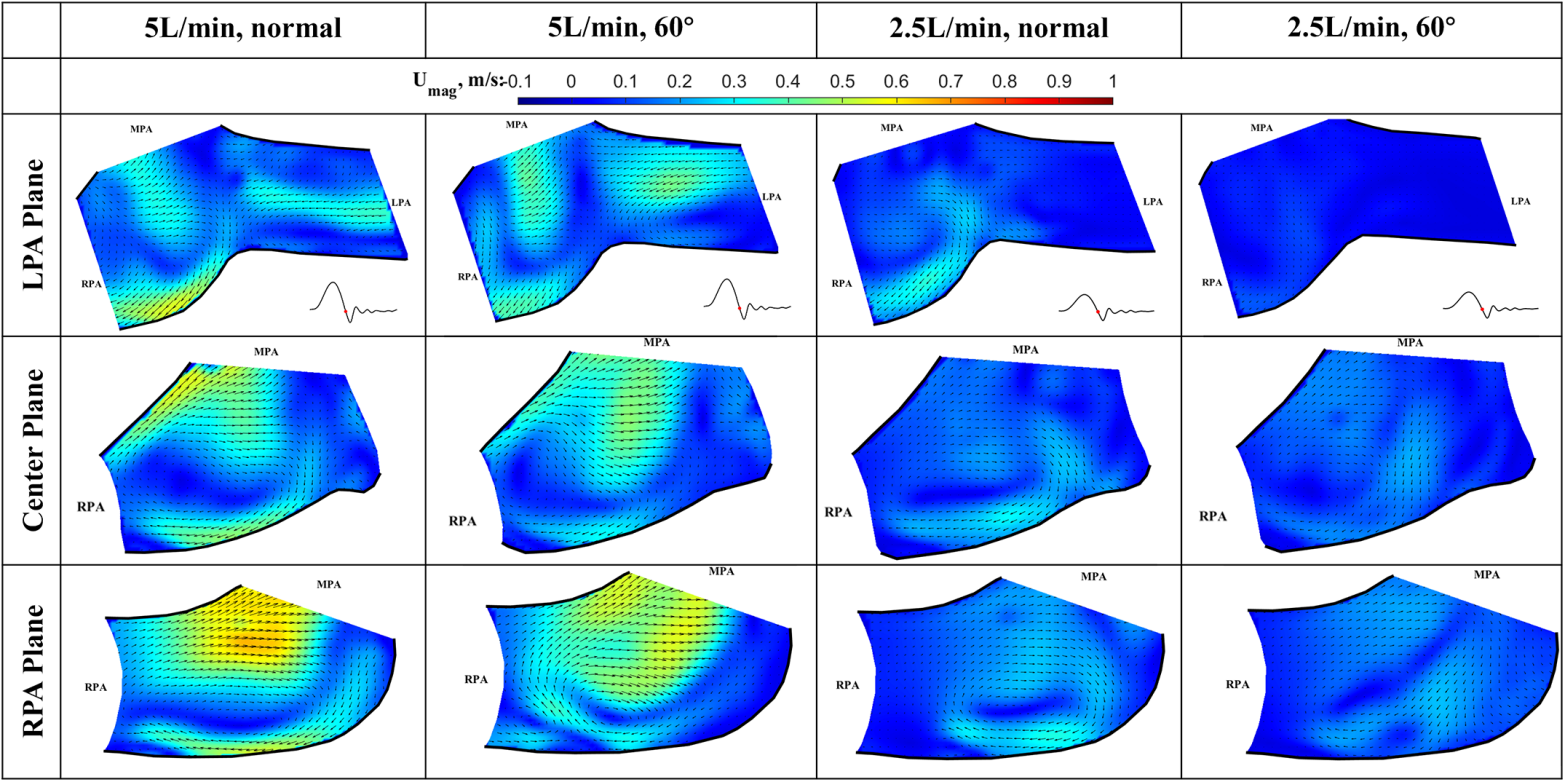
In-plane vector fields over contours of velocity magnitude during the deceleration phase for LPA/center/RPA planes (top/middle/bottom rows) for different flow rates and valve orientations. The time point corresponding to the flow phase in the cardiac cycle is shown in the insets in the first row.

**Figure 10:**
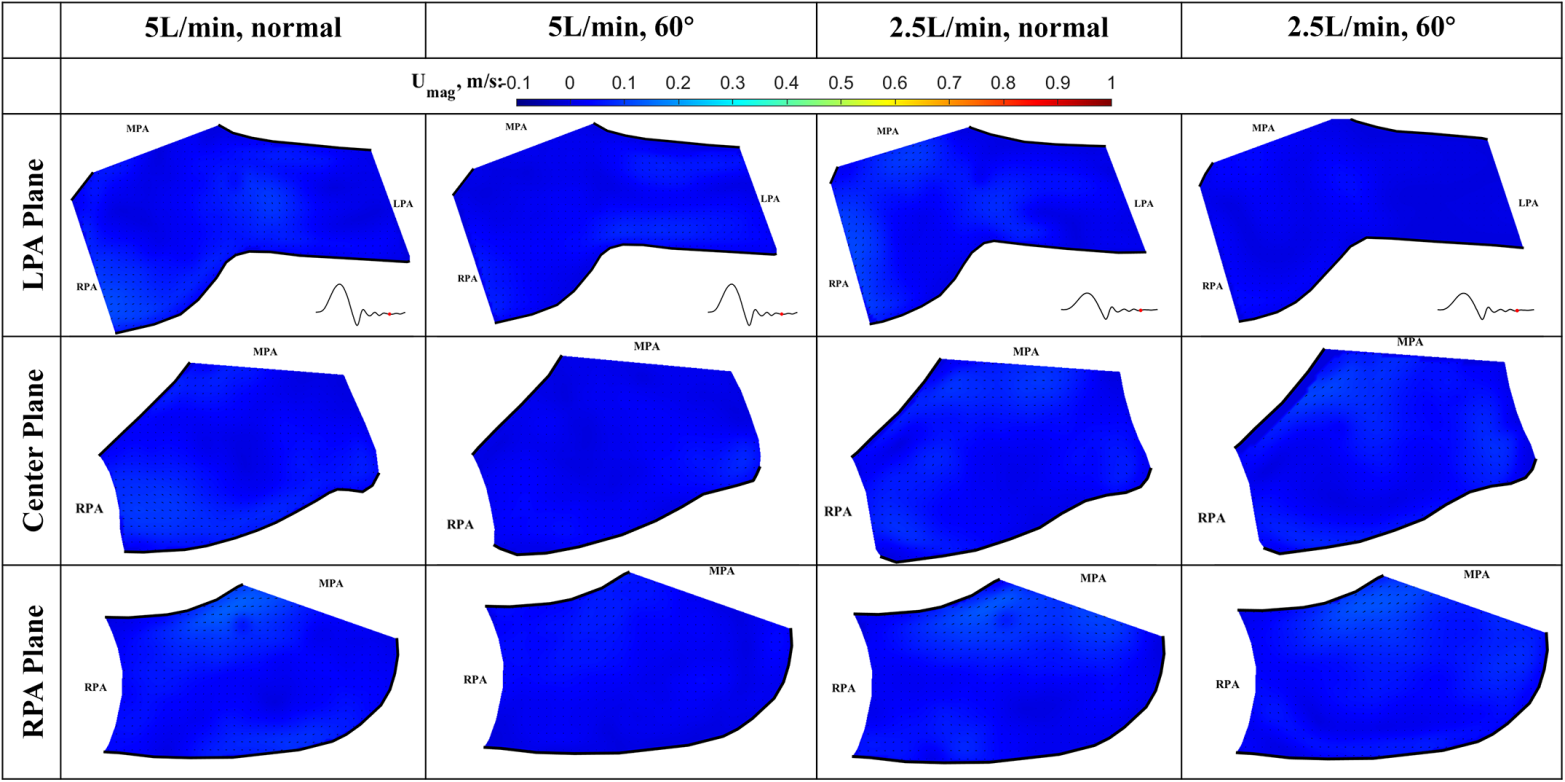
In-plane vector fields over contours of velocity magnitude at diastole for LPA/center/RPA planes (top/middle/bottom rows) for different flow rates and valve orientations. The time point corresponding to the flow phase in the cardiac cycle is shown in the insets in the first row.

The results of the flow visualization studies for the long valve (P36-30) are presented in Figures 7–10 according to the time point in the cardiac cycle, cardiac output, orientation, and the plane of visualization. The primary objective of visualizing the flow field is to assess the flow field downstream of the valve. Since there is an asymmetry between the arteries and the flow, the flow in the RPA and LPA planes was investigated independently. The LPA plane covers the center of the LPA and part of the RPA and is just downstream of the valve. Due to the existence of the long skirt, the flow in the vicinity of the leaflets could not be captured. In addition, the metallic stent blocks part of the laser and field of view of the cameras. While not affecting the majority part of the measurement, some artifacts are still visible. Notably the dark spots in Figures 7, 8.

The cardiac cycle is broadly divided into four phases—acceleration phase, peak systole, deceleration phase, and diastole. Data from the same time point will be discussed together, while data from the same plane are placed in the same row.

*Acceleration phase* (Figure 7): During the acceleration phase, the leaflets open, and flow rapidly accelerates towards the pulmonary artery bifurcation. The flow fields for different flow rates and valve orientations are all dominated by the newly formed jet from the RVOT. The forward pulmonary flow splits at the bifurcation and goes into both pulmonary arteries. There is no recirculation region observed in the LPA plane at this time point for all the cases, i.e., the flow in the LPA is being fully washed downstream.

When compared between different flow rates (2.5 L/min vs. 5 L/min) for the same valve orientation (normal and 60 deg), the shape of the main pulmonary jet is similar. However, the peak jet velocity magnitude at 2.5 L/min (around 0.6 m/s) is lower than that observed at 5 L/min (around 0.8 m/s). If compared across different valve orientations (normal vs. 60°) for the LPA plane, the main pulmonary jet appears much wider for the 60° case. This is related to the shape of the valve opening and the cut plane at different orientations. As shown by the definition of the LPA plane in Figure 3C and the valve opening in Figure 5, the valve opening is much wider for the 60° case at the LPA plane. As a result, the jet appears to be wider for the 60° case, while the peak velocity is similar for both valve orientations. On the contrary, the difference in the shape of the jet is small between the valves in the RPA plane. This has been evidently shown by the definition of the RPA plane in Figure 3C and the en face images in Figure 5, where the valve opening area is indeed similar for the two valve orientations at this plane.

*Peak systole* (Figure 8) At peak systole, the valve fully opens, and the forward flow reaches the peak flow rate. The flow field is dominated by a strong jet downstream of the valve for all the cases. The peak jet velocity reaches 0.95 m/s for the 5 L/min cardiac output and 0.56 m/s for the 2.5 L/min case. Similar to those shown in Figure 7 for the LPA plane, the shapes of the jets are different for different valve orientations and the jet downstream of the 60° oriented valve appears much wider. Again, this is consistent with the valve opening shape shown in Figure 5. The difference in the jet shape is much smaller for the Center and RPA planes. In the Center plane, a pair of counter-rotating vortices form at the edges of the main jet. The larger vortex near the RPA side corresponds to the same feature observed in the RPA plane. There is a stagnation point at the wall just downstream of the jet, but no flow separation is observed.

At a lower flow rate (2.5 L/min), the jets appear with lower magnitude, but the shape is similar to that for the higher flow rate. At both flow rates, the strong jet reaches the pulmonary bifurcation and splits into two, entering the LPA and RPA without flow separation. The much weaker jets at 2.5 L/min do not reach the far distal end of the RPA plane compared with the 5 L/min cases.

*Deceleration phase* (Figure 9): At the deceleration phase, the valve closes, and the main jet from the VenusP Valve System™ diminishes for all the cases. However, the remnant of the jet can be seen near the walls of the RPA. The remnant of the jet is more prominently shown in the RPA and Center planes. The recirculating vortex can be seen for both valve orientations and flow rates. Flow reversal is observed in the LPA at the high flow rate condition, which is a normal phenomenon during valve closing.

*Diastole* (Figure 10): During diastole, the flow is mostly quiescent. Low flow velocities are observed throughout the flow field for both valve orientations and flow rates.

### 3.4 Viscous shear stress at peak systole

**Figure 11:**
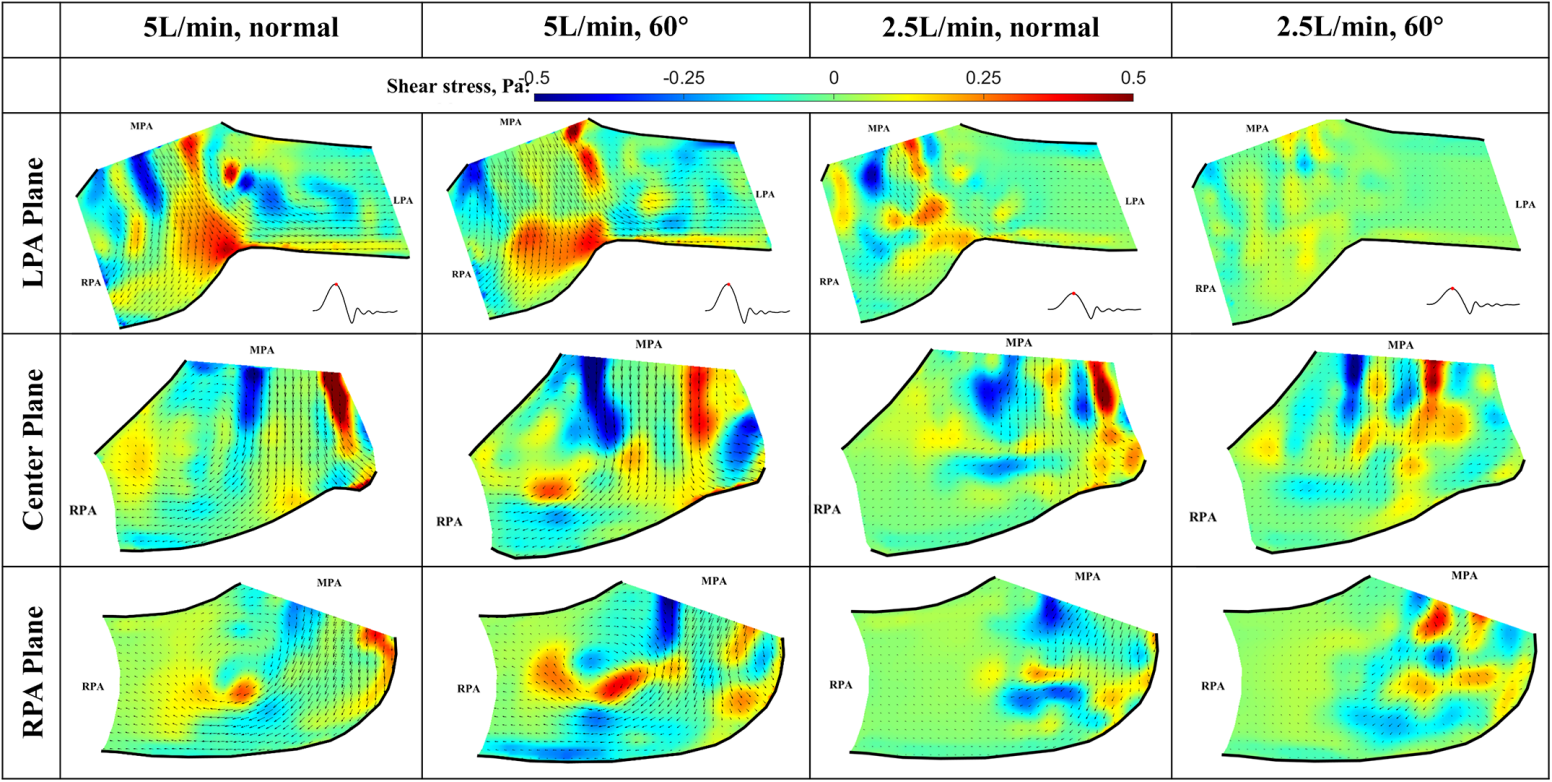
In-plane vector fields over contours of viscous stress at peak systole for LPA/center/RPA planes (top/middle/bottom rows) for different flow rates and valve orientations. The time point corresponding to the flow phase in the cardiac cycle is shown in the insets in the first row.

The viscous shear stress distributions during peak systole are shown in Figure 11. Based on the observation of the flow field, the shear stress is expected to peak at this phase for all flow conditions. The shear stress level is important in determining the risk of platelet activation and hemolysis (17–20). The viscous shear stress peaks in the shear layer between the main jet and the surrounding fluid, the values are highest shown in the Center plane. It is also elevated near the bifurcation where the main jet splits. However, the peak shear stresses for both flow rates and valve orientations are below 1 Pa, which is within the range of physiological shear stresses (Hathcock, 2006). Thus, we conclude that the viscous shear stress downstream of the valve will not cause platelet activation or hemolysis.

### 3.5 RSS at peak systole

**Figure 12:**
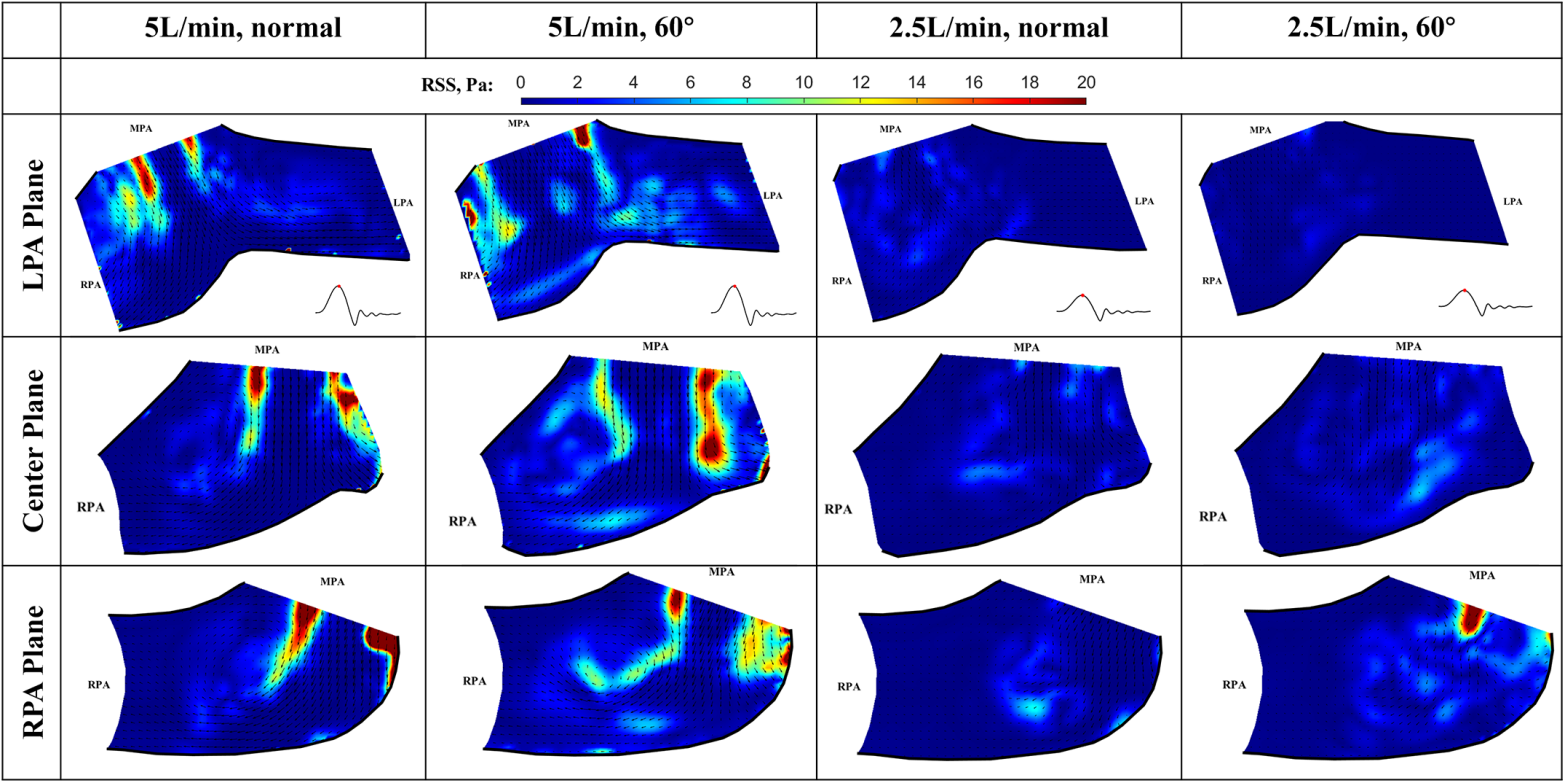
In-plane vector fields over contours of Reynolds shear stress at peak systole for LPA/center/RPA planes (top/middle/bottom rows) for different flow rates and valve orientations. The time point corresponding to the flow phase in the cardiac cycle is shown in the insets in the first row.

At peak systole, the valve fully opens, and the forward flow reaches its peak flow rate. As a strong vortex forms at the RPA side of the main jet, turbulence occurs in the shear layer between the forward jet and the quiescent surrounding fluid (Figure 12). The RSS is much higher for 5 L/min compared with 2.5 L/min cases. In the LPA plane, the peak RSS value for the normal valve at 5 L/min is around 21.6 Pa, while the peak RSS for the 60° oriented valve is around 28.1 Pa. In the RPA plane, at 5 L/min, the peak RSS is 81.2 Pa for the normal orientation and 46.0 Pa for the valve that is rotated by 60°. All the values are well below the blood damage threshold (21–23).

The RSS values for the low flow rate cases are significantly smaller. In the LPA plane, the peak RSS value for the normal and 60° oriented valves are 5.0 Pa and 3.5 Pa, respectively. Similarly, in the RPA plane, the peak RSS measured under 2.5 L/min at this plane is significantly higher (23 Pa) than that observed at the LPA plane.

### 3.6 RSS during deceleration

**Figure 13:**
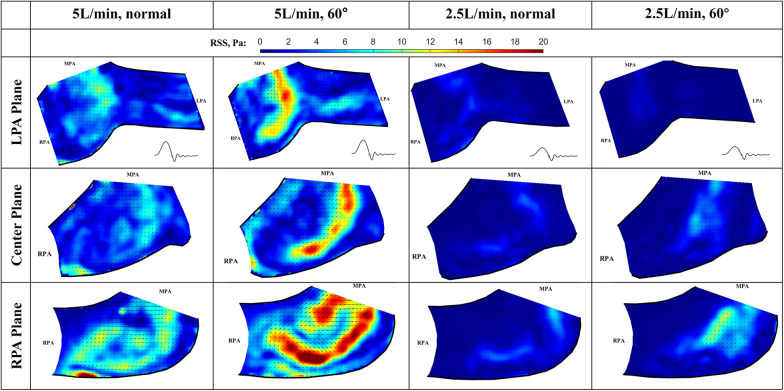
In-plane vector fields over contours of Reynolds shear stress during the deceleration phase for LPA/center/RPA planes (top/middle/bottom rows) for different flow rates and valve orientations. The time point corresponding to the flow phase in the cardiac cycle is shown in the insets in the first row.

At the deceleration phase, the valve is closing, and the pulmonary jet disappears in all cases, only the remnant of the jet can still be seen near the lower sidewall of the RPA. Interestingly, the turbulence level is high when the valve is closing, causing elevated levels of RSS in the remanent of the jet (Figure 13). The peak value at 5 L/min for the normal and 60° orientated valves are 12.5 Pa and 16.6 Pa, respectively. The RSS level is noticeably higher for the 60° orientated valve, indicating less flow stability compared with a normal-orientated valve. Again, the observed peak RSS is higher than that for the LPA plane. At 5 L/min and in the RPA plane, the peak values for the normal and 60° orientated valves are 22.9 Pa and 23.4 Pa, respectively. The peak RSS is higher for the 60° orientated valve, which is consistent with the observations for the LPA plane.

For the low flow rate at 2.5 L/min in the LPA plane, the peak values for the normal and 60° orientated valves are 3.7 Pa and 1.5 Pa, respectively. As expected, both values are significantly lower when compared with the high flow rate cases. In the RPA plane, the peak values for the normal and 60° orientated valves are 6.6 Pa and 10.3 Pa, respectively. The consistent trend shows that the RSS value is higher at the RPA plane for all the flow rates and valve orientations.

## 4 Discussion and conclusion

Hydrodynamic performance testing, high-speed en face imaging of leaflet motion, and phase-locked and high-speed Particle Image Velocimetry (PIV) experiments were conducted on two 36 mm VenusP Valve System™ pulmonary valves to assess their detailed fluid dynamic characteristics. Experiments were mostly conducted in accordance with the ISO 5840-1-2021 standard, except for the imaging plane selection. En face videos are used to analyze the kinematics of the leaflets during a cardiac cycle, the phase-locked PIV provides high-resolution mean flow fields, and the high-speed PIV gives us a comprehensive view of the flow variations during a cardiac cycle. The two valves of different lengths were evaluated at 70 BPM and two cardiac output conditions: 5 L/min and 2.5 L/min. For the long valve (P36-30), two different valve orientations were studied: the normal orientation when the two leaflet commissures are aligned with both pulmonary arteries and the 60° orientation when the valve was rotated 60° in the counter-clockwise direction. Phase-locked PIV and high-speed PIV data were acquired for the long valve at three spatial planes (LPA, RPA, and Center planes) to study the three-dimensional extent of flow downstream of the valve and in the pulmonary arteries. For both valves, the hemodynamic performance was evaluated by assessing the transvalvular pressure gradient and effective orifice area of the valve. The geometric orifice area was calculated from the high-speed en face videos.

PIV measurements revealed the detailed flow fields downstream of the valve and in the pulmonary arteries. At early systole, the newly formed main jet from the valve pushes the flow field downstream of the LPA and RPA. When the jet is fully developed at peak diastole, it splits at the bifurcation and enters both PAs. A large vortex forms at the edge of the jet at the RPA side. The jet diminishes, and the vortex grows during the deceleration phase. The flow field becomes quiescent at diastole. The formation of a recirculating vortex is normal when a strong jet enters the quiescent flow. The vortex formation does not represent flow stasis, as the flow is being washed downstream at early systole. The flow phenomena observed across different flow rates for the same valve orientation are similar, except that the velocity magnitude is lower for the low flow rate case. However, the flow phenomena for the same flow rate but different valve orientations are not the same, as the shape of the jet is determined by the shape of the valve opening. No significant differences in hemodynamic performances are observed for all the valves and orientations. Detailed analyses of the flow fields obtained by both phase-locked and high-speed PIV measurements show no visible flow stasis within the measured planes. Furthermore, the peak Reynolds shear stress is well below the threshold level for blood damage for all cases. Although higher RSS was observed in the RPA plane for the 60° orientated valve when compared with other configurations, it should not be a concern for hemolysis due to the low peak values. For all cases, the viscous shear stresses at peak systole are still within physiological stress ranges. Therefore, we conclude that the risk of hemolysis and thrombosis is low for both valves and configurations. More detailed analyses of flow stasis are included in the parallel computational fluid dynamics studies based on 3D flow fields.

### 4.1 Limitation

The primary limitation of this study is the *in vitro* nature. Firstly, the right heart simulator used in the study was made with rigid tubing and adjusted by compliance and resistance elements, while the human pulmonary artery is flexible, which affects the pressure and flow and the valve deployment. In addition, the pulmonary arteries were idealized based on the mean values of the measured patient data. However, since this study is to evaluate the performance of the valve, and the valve was corrected and deployed, the use of an idealized and rigid model won't affect the key findings of the paper. Secondly, the hemodynamic environment does not cover all the flow ranges recommended by the ISO 5840 standard, but it provides sufficient information to evaluate the valve performance. Thirdly, the PIV measurement was in 2D, while the pulmonary flow is inherently 3D, which will be studied in our future work. Fourthly, the blood analog used in the study was Newtonian, while the blood is a non-Newtonian fluid. However, since we are focused on the flow in the large arteries, the non-Newtonian effect of the blood is unlikely to cause major changes to the flow. Fifth, the pressures in the PAs were measured distal to the bifurcation, and due to pressure loss in the bifurcation, the calculated EOA differed from that with the pressure measured just downstream of the valve. Since the pressures might be measured at different locations across different studies, caution should be taken when comparing the results. Finally, only one valve of each size was tested; however, these valves are expected to be reproducible, and thus, the results should be representative.

## Data Availability

The raw data supporting the conclusions of this article will be made available by the authors, without undue reservation.
