# Supplementary material for: Hemodynamics of the VenusP Valve System™—an in vitro study
Source: Front Med Technol. 2024 May 2;6:1376649. doi: 10.3389/fmedt.2024.1376649 (PMC11098565; doi:10.3389/fmedt.2024.1376649)
Supplement: Supplementary file 1 [file Datasheet1.docx]

Supplementary Material


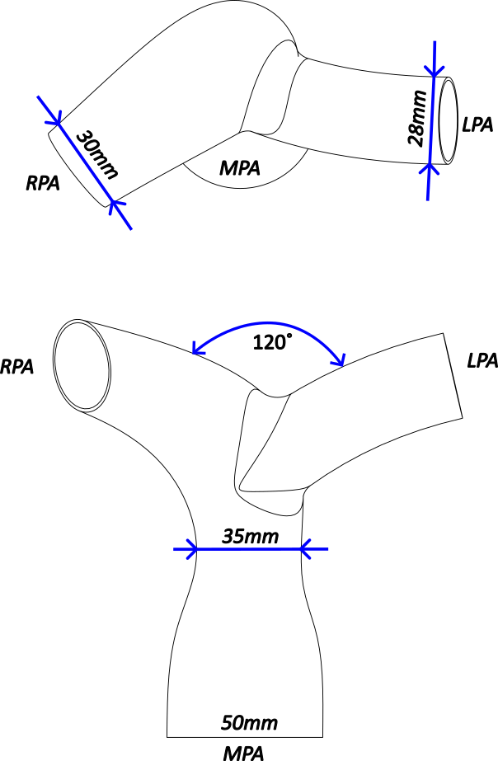


**Supplementary Figure 1.** The geometry of the idealized pulmonary artery model.
